# Rapid host adaptation by extensive recombination

**DOI:** 10.1099/vir.0.007724-0

**Published:** 2009-03

**Authors:** Eric van der Walt, Edward P. Rybicki, Arvind Varsani, J. E. Polston, Rosalind Billharz, Lara Donaldson, Adérito L. Monjane, Darren P. Martin

**Affiliations:** 1 Department of Molecular and Cell Biology, University of Cape Town, Cape Town, South Africa; 2 Institute of Infectious Disease and Molecular Medicine, University of Cape Town, Cape Town, South Africa; 3 Electron Microscope Unit, University of Cape Town, Cape Town, South Africa; 4 Department of Plant Pathology, University of Florida, Gainesville, FL 32611, USA; 5 Department of Microbiology, University of Washington, Seattle, Washington, USA

## Abstract

Experimental investigations into virus recombination can provide valuable insights into the biochemical mechanisms and the evolutionary value of this fundamental biological process. Here, we describe an experimental scheme for studying recombination that should be applicable to any recombinogenic viruses amenable to the production of synthetic infectious genomes. Our approach is based on differences in fitness that generally exist between synthetic chimaeric genomes and the wild-type viruses from which they are constructed. In mixed infections of defective reciprocal chimaeras, selection strongly favours recombinant progeny genomes that recover a portion of wild-type fitness. Characterizing these evolved progeny viruses can highlight both important genetic fitness determinants and the contribution that recombination makes to the evolution of their natural relatives. Moreover, these experiments supply precise information about the frequency and distribution of recombination breakpoints, which can shed light on the mechanistic processes underlying recombination. We demonstrate the value of this approach using the small single-stranded DNA geminivirus, maize streak virus (MSV). Our results show that adaptive recombination in this virus is extremely efficient and can yield complex progeny genomes comprising up to 18 recombination breakpoints. The patterns of recombination that we observe strongly imply that the mechanistic processes underlying rolling circle replication are the prime determinants of recombination breakpoint distributions found in MSV genomes sampled from nature.

## INTRODUCTION

It has long been recognized that recombination is important for its role in unlinking deleterious mutations from those that may be neutral or beneficial (Felsenstein, 1974; Keightley & Otto, 2006), thus allowing populations at least partial escape from the negative fitness effects of accumulating slightly deleterious mutations (Chao, 1990; Muller, 1964). Similarly, recombination allows otherwise asexual organisms such as viruses and bacteria to avoid the evolution-retarding effects of ‘clonal interference’, which results from competition among distinct beneficial mutations that reside concurrently in multiple genomes – eventually one dominates within the population at the expense of the rest (Gerrish & Lenski, 1998; de Visser & Elena, 2007).

In viruses, where genetic exchange between different species or even unrelated taxa is possible, recombination is also capable of generating spectacular genetic diversity. While natural recombination between distantly related genomes has only rarely been shown to occur in double-stranded DNA and RNA viruses (Suzuki *et al.*, 1998; Varsani *et al.*, 2006), it is apparently quite common amongst most reverse-transcribing (Chenault & Melcher, 1994; Katz & Skalka, 1990; Sharp *et al.*, 1995), positive-sense single-stranded RNA (Chare & Holmes, 2006; Heath *et al.*, 2006) and single-stranded DNA (ssDNA) (Heath *et al.*, 2004; Padidam *et al.*, 1999; Shackelton *et al.*, 2007; Worobey, 2000) viruses.

Such inter-species recombination features particularly prominently in the evolution of ssDNA geminiviruses (Delatte *et al.*, 2005; García-Andrés *et al.*, 2006, 2007a; Lefeuvre *et al.*, 2007a, b; Ndunguru *et al.*, 2005; Owor *et al.*, 2007a; Padidam *et al.*, 1999; Prasanna & Rai, 2007; Shepherd *et al.*, 2008a; Varsani *et al.*, 2008a, b; Zhou *et al.*, 1998). While bioinformatic analyses of naturally occurring inter-species recombinants have dominated the geminivirus literature, three reports have indicated that geminivirus recombination either occurs naturally or can be induced to occur within time-frames that make it amenable to analysis by direct experimental approaches (García-Andrés *et al.*, 2007b, Stenger *et al.*, 1991; Schnippenkoetter *et al.*, 2001). Most notably, these studies definitively demonstrated that the virion strand origin of replication (v*-ori*) is a recombination hot-spot, a fact that has only recently been statistically verified using geminivirus genomes sampled from nature (Lefeuvre *et al.*, 2007a; Varsani *et al.*, 2008b).

Maize streak virus (MSV), the type strain of the genus *Mastrevirus* in the family *Geminiviridae*, has a simple genome consisting of virion sense movement protein (*mp*) and coat protein (CP) (*cp*) genes, and a complementary sense replication-associated protein (*rep*) gene that is expressed in two alternatively spliced isoforms. Separating the complementary and virion sense genes are a long intergenic region (LIR), containing the v-*ori* and transcriptional promoter elements, and a short intergenic region (SIR), containing the complementary sense *ori* and transcription termination elements.

Here, we use a conceptually simple but powerful new experimental system to demonstrate how recombination in geminiviruses such as MSV can be a remarkably efficient mechanism capable of rapidly generating progeny genomes with increased fitness.

## METHODS

### Viruses.

Agroinfectious wild-type isolates MSV-MatA (Martin *et al.*, 2001) and MSV-VW (Willment *et al.*, 2002) and chimaeric MatMPCPVW and VWMPCPMat clones (Martin & Rybicki, 2002) have been described previously. Maize samples field-infected with the wild-type isolates MSV-MakD and MSV-Gat have been described previously (Martin *et al.*, 2001). Maize leaf material field-infected with the MSV-A isolates, MSV-Gre and MSV-Mit, was obtained from Greytown, South Africa, in 2002 and Mitungu, Kenya, in 1998, respectively.

### System for generating recombinant viruses.

We have previously characterized several MSV reciprocal chimaeras, one pair of which, MatMPCPVW and VWMPCPMat, were chosen to illustrate our proposed scheme for studying recombination. These chimaeras were constructed from two wild-type MSV genomes: MSV-MatA, a typical maize-adapted MSV-A isolate and MSV-VW, a typical *Digitaria*-adapted MSV-B isolate (Varsani *et al.*, 2008b). Although these isolates are quite similar (they share 89 % genome-wide sequence identity) MSV-VW is less pathogenic in maize. MatMPCPVW and VWMPCPMat are chimaeric MSV genomes with precise exchanges of both virion-sense open reading frames between MSV-MatA and MSV-VW. In maize, both these chimaeras are more pathogenic than MSV-VW, but less pathogenic than MSV-MatA. In particular, the chimaeras barely produce symptomatic infections in the MSV-resistant maize genotype PAN6099, whereas MSV-MatA produces moderately severe infections in this host (Martin & Rybicki, 2002).

As MatMPCPVW and VWMPCPMat collectively contain the complete genomic sequence of MSV-MatA, it could be expected that they might optimally recombine to produce MSV-MatA-like maize-adapted daughter genomes. It was helpful that the chimaeric viruses rarely produce symptomatic infections in MSV-resistant maize genotypes because the appearance of severe symptoms in co-infected MSV-resistant plants served as an indicator of recombination having yielded maize-adapted daughter genomes. A key feature of this experimental design was that we knew in advance that at least one fit, maize-adapted MatMPCPVW–VWMPCPMat recombinant was possible: MSV-MatA. We could therefore compare the genetic distance and fitness of any recombinants that emerged with this ‘target recombinant solution’.

### Agroinoculation, leafhopper transmission and screening of plants.

Three-day-old PAN6099 seedlings were agroinoculated as described by Martin *et al.* (1999) with mixtures of equal volumes of MatMPCPVW- and VWMPCPMat-containing *Agrobacterium tumefaciens* cultures. Batches of ∼100 seedlings were agroinoculated in each of four experiments. Between 60 and 80 days after inoculation, DNA was isolated from all plants displaying symptoms of infection.

Alternatively, 3-day-old MSV-sensitive sweetcorn seedlings (either cultivar Golden Bantam or Jubilee) were co-infected with MatMPCPVW and VWMPCPMat and maintained for 30 days before transmitting viruses from them into 8-day-old MSV-resistant maize seedlings (either PAN6099 or PAN77). Between six and ten *Cicadulina mbila* leafhoppers caged on individual plants were used for these transmissions, which were carried out according to the method described by Shepherd *et al.* (2007).

### Isolation, cloning, screening and sequencing of viral DNA.

Viral DNA was cloned from infected plant tissue either by using previously described methods (Palmer *et al.*, 1998; Inoue-Nagata *et al.*, 2004; Owor *et al.*, 2007b; Shepherd *et al.*, 2008b) or via an isopycnic gradient centrifugation method. Leaf material (0.5–2 g) was ground under liquid nitrogen and mixed with 6 ml extraction buffer [0.1M Tris/HCl, 0.1M NaCl, 0.1M EDTA, 1 % (w/v) SDS, pH 7]. Following 10 min of shaking, debris was pelleted at 5000 ***g*** for 10 min at 20 °C. CsCl (5.5 g) was dissolved in 5.5 ml supernatant. Ethidium bromide (400 μl of a 10 mg ml^−1^ solution) and 1 μg pBluescriptSK+ (Stratagene) were added to 5 ml supernatant. This was centrifuged at 275 000 ***g*** for 16 h at 20 °C. The lower of two DNA bands visible under 310 nm UV light was collected and extracted twice with NaCl-saturated 2-propanol and twice with 70 % ethanol (Sambrook *et al.*, 1989). The entire DNA pellet was resuspended in the appropriate buffer, digested with *Bam*HI, reprecipitated with ethanol, ligated with T4 ligase and used to transform competent *Escherichia coli* DH5*α* according to standard protocols (Sambrook *et al.*, 1989). White colonies on X-Gal-IPTG Luria agar plates were tested for the presence of MSV DNA using the colony hybridization protocol described in the Hybond-N+ manual (GE Healthcare), using a DIG-labelled MSV-A DNA probe (Roche), as described by Willment *et al.* (2007).

Universal M13 sequencing primers were used to amplify plasmid inserts by PCR (Gussow & Clackson, 1989) from all colonies containing virus DNA. Restriction fragment length polymorphism (RFLP) analysis (Willment *et al.*, 2001) identified potentially unique recombinant genomes and subgenomes which were sequenced by primer walking (Owor *et al.*, 2007b). All sequences are available on request from the authors.

### Construction of infectious recombinant progeny virus clones and assessment of virulence.

Agroinfectious clones were constructed in pBI121 (Martin & Rybicki, 2000) and their virulence was quantified in MSV-sensitive (cv. Jubilee) and -resistant (PAN6099) maize genotypes using image analysis of chlorotic leaf areas (Martin & Rybicki, 1998; Martin *et al.*, 1999).

## RESULTS

### Adaptive recombination *in planta* between two defective MSV genomes

We agroinoculated approximately 400 MSV-resistant maize plants with mixed MatMPCPVW and VWMPCPMat inocula, and identified 30 that unambiguously developed symptoms ranging from extremely mild (a few small stippled chlorotic streaks per leaf) to moderately severe (long continuous chlorotic streaks covering up to 40 % of the leaf area). To remove any doubts regarding PCR- or rolling circle amplification-induced mutations (Ge *et al.*, 2007) and/or recombination (Odelberg *et al.*, 1995), we cloned MSV genomes directly using CsCl gradient-purified DNA from 12 of these plants. Between 30 and 100 MSV DNA-containing clones (determined by colony hybridization) were characterized from each plant by colony PCR and RFLP analysis (data not shown). Whereas six mildly symptomatic plants apparently contained only non-recombinant parental virus genomes, six moderately symptomatic plants contained what appeared to be full-length recombinant genomes. This analysis also indicated that we had cloned a surprisingly large number of subgenome length MSV DNA fragments, which are described in greater detail below. We were unable to clone full-length MSV genomes from the remaining plants.

**Fig. 1. f1:**
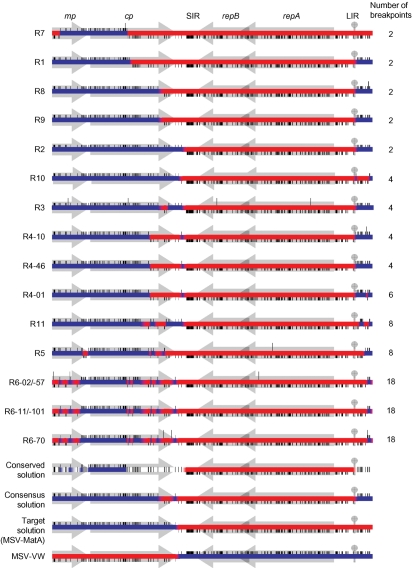
Experimental MSV recombination patterns. The consensus recombinant solution (the parent from which >50 % of sites are derived), the conserved solution (the parent from which 100 % of sites are derived) and wild-type MSV genomes (MSV-MatA and MSV-VW) are shown for comparative purposes. Short vertical lines above or below the centre line denote VWMPCPMat- and MatMPCPVW-derived polymorphisms, respectively. Red and blue, respectively, indicate MSV-MatA- and MSV-VW-derived sequences. Long vertical lines represent point mutations. Positions of genes (*mp*, *cp* and *repA+repB*), intergenic regions (LIR and SIR) and the v-*ori* (circle) are indicated in shaded grey.

Depending on the number of unique RFLP patterns detected, complete genome sequences were determined for between one and five full-length clones from each of the 12 symptomatic MSV-resistant plants from which we were able to retrieve full-length genomes. This sequence data confirmed that the six very mildly symptomatic plants contained either MatMPCPVW or VWMPCPMat sequences (never both together) and demonstrated that the six plants displaying moderate MSV symptoms contained MatMPCPVW–VWMPCPMat recombinants (Fig. 1, recombinants R1–R6).

Given the low efficiency with which MatMPCPVW and VWMPCPMat symptomatically infect MSV-resistant maize genotypes such as PAN6099 (6.2 and 9 %, respectively; Martin & Rybicki, 2002), it is not surprising that recombinants were only retrieved from approximately 1.5 % of co-inoculated plants. We therefore sought to improve our methods to increase the efficiency with which recombinants were retrieved. We ultimately made two modifications. First, we generated MatMPCPVW–VWMPCPMat co-infections in an MSV-sensitive maize genotype (sweetcorn cv. Golden Bantam) and used leafhoppers to transmit the resulting mixed infections to MSV-resistant maize plants (genotypes PAN6099 or PAN77), in order to select for maize-adapted recombinants. Second, we used phi29 DNA polymerase to perform rolling circle amplification before cloning full-length genomes. We co-infected ∼80 sweetcorn plants with MatMPCPVW and VWMPCPMat and 30 days later, we used leafhoppers to transmit viruses from 17 of the most severely infected plants to uninfected MSV-resistant maize. Five of the MSV-resistant plants developed moderately severe symptoms within 30 days and an additional five full-length recombinant genomes were isolated and sequenced from these plants (Fig. 1, sequences R7–R11).

### Recombinants display a range of unique and often complex mosaics

Ten unique recombinants, each from a different plant, were identified among the 17 sequenced clones that we analysed (Fig. 1). Recombinants R8 and R9 displayed identical mosaic structures, even though they were isolated from different plants. Minor variations detected amongst sequences isolated from individual plants by PCR-RFLP analysis consisted of point mutations and/or small recombination events (see the five R6 and three R4 recombinants in Fig. 1).

In general, two major classes of recombinants were identified. In the first and simplest of these, comprising eight of the eleven recombinants and containing between two and six recombination breakpoints each (R1–R4 and R7–R10 in Fig. 1), the recombinant genomes essentially consisted of fusions of VWMPCPMat virion-sense sequences with MatMPCPVW complementary-sense sequences. In these genomes, one breakpoint invariably occurred either at the v-*ori* (7/8 recombinants) or within 300 nt of v-*ori*, with another breakpoint occurring in either the SIR or the 3′ half of *cp*.

A second class of recombinants (R5, R6 and R11) contained between eight and 18 breakpoints, none of which occurred precisely at the v-*ori*. As with the simple recombinants, all the breakpoints in this second group fell within the virion-sense coding half of the genome.

### Recombination does not appear to be associated with increased mutation rates

It is noteworthy that eight of the 17 sequenced recombinant genomes contained one or more mutations. A total of 17 mutations were observed, giving an average mutation frequency of 3.76×10^−4^ substitutions per site or, given that each experiment lasted about 2 months, a mutation rate of 2.3×10^−3^ substitutions per site per year. Although this mutation rate is approximately three times higher than those reported in experimental studies of MSV evolution (Isnard *et al.*, 1998; van der Walt *et al.*, 2008), it is probably biased upwards because sequences were selected for analysis based on RFLP analyses. Despite this expected bias, the rate is very similar to that estimated over a similar time-frame for the related geminivirus tomato yellow leaf curl China virus (Ge *et al.*, 2007). Therefore, it is unlikely that the mutations we detected are causally linked to the process of recombination.

### The recombination breakpoint distribution approximates that seen in nature

**Fig. 2. f2:**
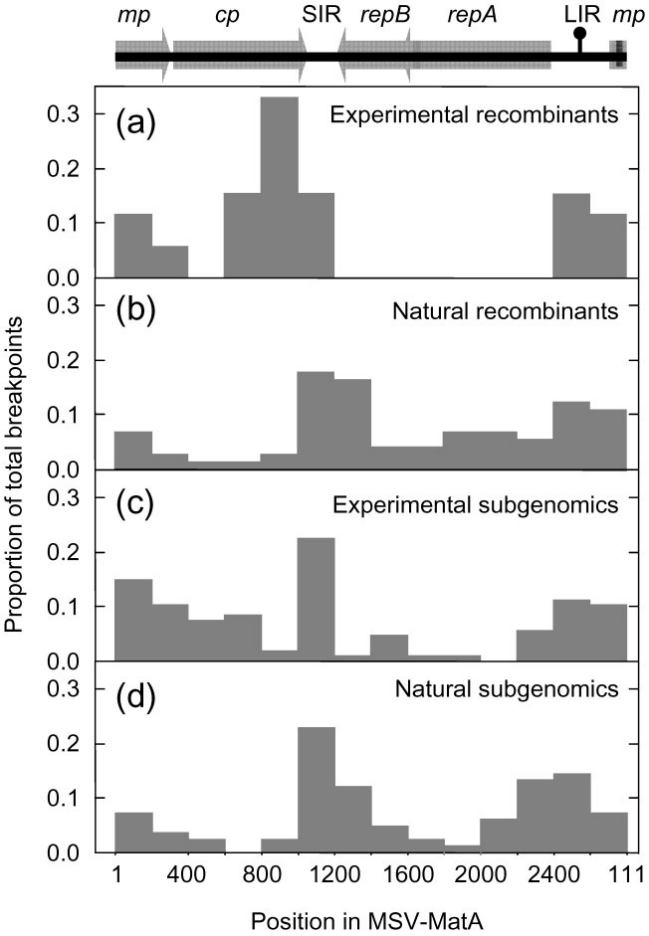
Recombination breakpoints are not randomly distributed. (a) Distribution of 53 homologous recombination breakpoints within experimentally observed recombinants. (b) Distribution of 73 homologous recombination breakpoints observed in natural MSV recombinants (Varsani *et al.*, 2008b). (c) Distribution of 107 non-homologous recombination breakpoints detected in subgenomic sequences sampled during recombination experiments. (d) Distribution of 83 non-homologous recombination breakpoints detected in subgenomic sequences sampled from natural MSV infections. Genome regions indicated above the plots are labelled as in Fig. 1. Nucleotides 1–111 are represented in both the first and last bars of the histograms because the MSV genome length is not an exact multiple of 200.

The 52 recombination breakpoints we detected were clearly non-randomly distributed. None were detected in the complementary sense genes; however, those we did observe tended to occur most frequently around the v-*ori* and in the 3′ half of *cp* and the SIR (Fig. 2a[Fig f2]). This distribution is similar, but not identical, to that reported recently for recombination breakpoints in natural MSV recombinants (Varsani *et al.*, 2008b; Fig. 2b[Fig f2]). While both distributions indicate that breakpoints occur with increased frequency around the v-*ori*, the breakpoint cluster spanning the 3′ portion of *cp* and the SIR is shifted by approximately 200 nt between the two distributions. Also, none of the recombinants analysed here had breakpoints within the replication associated gene (*rep*/*repA*), whereas an appreciable number of natural MSV recombinants have breakpoints in this region (Varsani *et al.*, 2008b; Owor *et al.*, 2007a).

### The recovered recombinant genomes were fitter than their parents

To test whether the detected recombination events were adaptive and had yielded genomes with increased fitness in maize, we compared the virulence of six recombinants (R6-02, R6-11 and R6-101 from plant P6, R4-01 and R4-10 from plant P4 and R1 from plant P1) with that of their parents (MatMPCPVW and VWMPCPMat) and the wild-type viruses, MSV-MatA (the assumed ‘target’ recombinant solution) and MSV-VW. We examined these 10 viruses in both MSV-sensitive and -resistant maize genotypes using quantified chlorotic leaf areas as an approximation of viral fitness (Martin *et al.*, 2005a).

**Fig. 3. f3:**
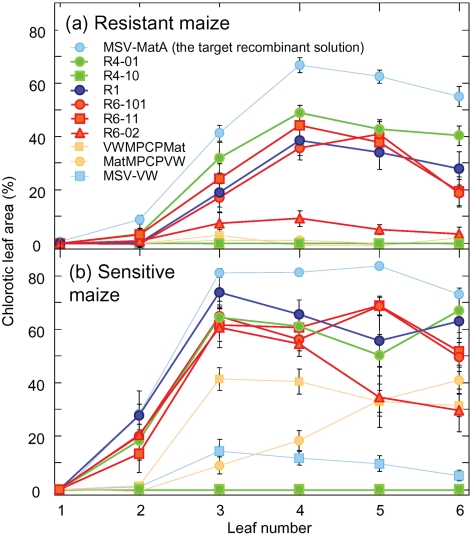
Recovered recombinants are generally fitter in maize than their inoculated chimaeric parents. Proportion of chlorotic areas occurring on leaves 2–6 of PAN6099 (MSV-resistant) (a) and cv. Golden Bantam (MSV-sensitive) (b) plants agroinoculated with recombinant (R), parental chimaera (MatMPCPVW and VWMPCPMat) and wild-type (MSV-MatA and MSV-VW) MSV genomes. Symptoms on leaves 2, 3, 4, 5 and 6 were recorded 15, 15, 22, 29 and 36 days post-inoculation, respectively.

In both MSV-sensitive and -resistant hosts, all but one of the recombinants (R4-10) resulted in more severe symptoms than MatMPCPVW, VWMPCPMat and MSV-VW (Fig. 3). R4-10 failed to produce symptoms in either host and R6-02 was considerably less virulent than the other two viruses from plant P6. Both R6-02 and R4-10 carry point mutations which are probably responsible for their reduced virulence, because these mutations differentiate them from very similar but more pathogenic viruses sampled from the same plants (Fig. 1).

### Recombinants tended to resemble the target recombinant solution

**Fig. 4. f4:**
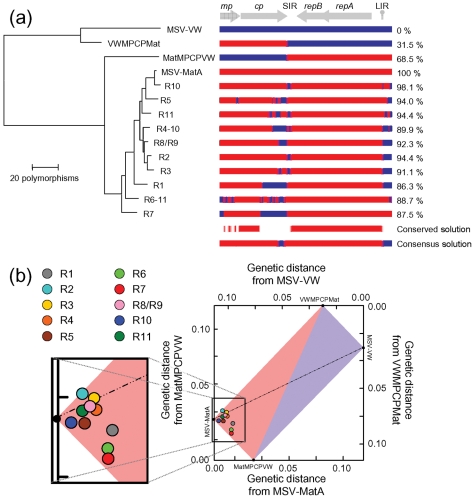
Convergence of recombinants on the target recombinant solution. (a) Dendrogram graphically depicting the convergence of recombinants (R1–R11) on the target solution (MSV-MatA). Red and blue, respectively, indicate MSV-MatA- and MSV-VW-derived sequences. The percentage of MSV-MatA-derived polymorphisms occurring in each recombinant is shown to the right. Genome regions (above) are labelled as in Fig. 1. (b) A Hamming distance graph illustrating that recombinant genomes resemble MSV-MatA. Each point represents one recombinant sequence with the positions of parental chimaeras, MSV-MatA and MSV-VW indicated on the axes.

We examined the efficiency with which recombination achieved the expected ‘target solution’, MSV-MatA. To achieve a ‘perfect’ result by this standard (i.e. the recreation of MSV-MatA), recombination would have had to reunite all 248 MSV-MatA-derived polymorphisms within a single genome. The recombinants that we sequenced contained between 86.3 (R1) and 98.1 % (R10) of these MSV-MatA-derived polymorphisms and were therefore all much more closely related to MSV-MatA than they were to either their immediate parents or MSV-VW (Fig. 4).

Although only MSV-MatA-derived polymorphisms were absolutely conserved among all of the recombinants (see the ‘conserved solution’ in Fig. 4a[Fig f4]), most recombinant genomes retained small regions of MSV-VW sequence between the v-*ori* and the start of *mp*, and in the 3′ portion of *cp* and the 5′ portion of the SIR (see the ‘consensus solution’ in Fig. 4a[Fig f4]). These two regions coincide with the sites at which MSV-MatA and MSV-VW sequences were ligated during construction of MatMPCPVW and VWMPCPMat.

### Homologous and non-homologous breakpoint distributions are similar

**Fig. 5. f5:**
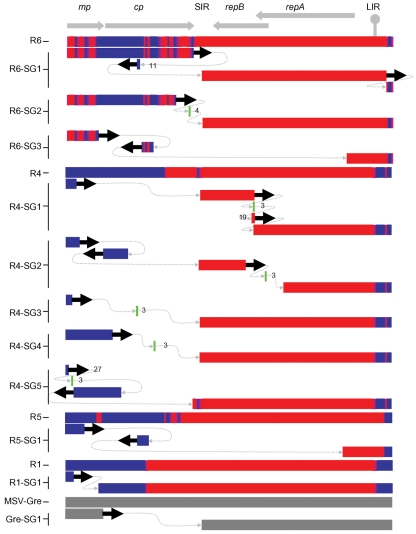
The structures of MSV subgenomics are often complex. Examples of subgenomic sequences are presented along with their full-length parental genomes. Discrete sequence elements are presented on different lines with dotted grey lines indicating the order in which they were arranged and thick black arrows indicating their orientation. MatMPCPVW- and VWMPCPMat-derived sequences are given in red and blue, respectively; the MSV-Gre subgenomic from a natural maize infection is shown in grey. Sequence elements too short to identify are in green. The length of elements shorter than 50 bp are given. Genome regions (above) are labelled as in Fig. 1.

Analysis of our MSV genomic DNA libraries revealed that more than 70 % of the cloned MSV fragments from all plants were smaller than the full genome. Sixteen complete DNA sequences and a further 12 partial sequences of these ‘subgenomics’ revealed genomic rearrangements including large deletions, translocations, inversions and sequence duplications (Fig. 5). Short stretches of between 1 and 4 nt of unconfirmed origin were found between some adjacent blocks of rearranged MSV sequence, but many junctions clearly consisted of precise transitions from one MSV-derived sequence segment to another.

As we had specifically isolated circular DNA and none of the sequences contained internal *Bam*HI restriction sites (the enzyme used for cloning), it is unlikely that these subgenomics are simply cloning artefacts. However, it was apparent that at least some of them were incomplete fragments of larger molecules found within plants. This was evident from the observation that inverted repeat sequences carrying the conserved *Bam*HI site were found on the ends of some cloned fragments. This arrangement would be expected if the clone was derived from a larger fragment carrying an inverted repeat of the MSV genome region carrying the *Bam*HI site. Similarly, it is possible that other subgenomic clones that we characterized may also have been derived from larger concatamers of MSV DNA.

Although similar MSV subgenomics have been described in natural MSV infections (Casado *et al.*, 2004), we suspected that the conditions of our co-infection experiments may have stimulated their occurrence. However, when we examined four MSV-A natural field infections, we found similar ratios of subgenomic to full-length sequences and similar distributions of non-homologous recombination breakpoints within 18 complete and eight partial sequences of subgenomic clones (compare Fig. 2c and d[Fig f2]). We also found similar ratios of subgenomics to full-length genomes regardless of whether we isolated them using our isopycnic gradient ultracentrifugation method or a more conventional alkaline lysis-based approach (data not shown).

**Fig. 6. f6:**
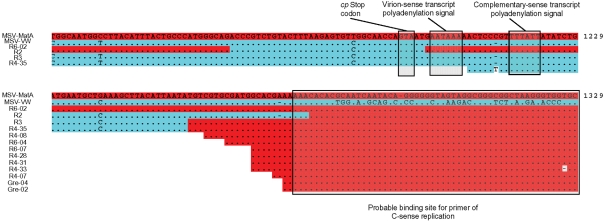
Recombination breakpoints within the SIR. Identity with MSV-MatA is indicated with full-stops, gaps introduced to optimize the alignment are indicated with dashes and coordinates are relative to the last A residue of the MSV-MatA v*-ori* nonanucleotide. Red and blue, respectively, indicate MSV-MatA- and MSV-VW-derived sequences.

Strikingly, several subgenomics had non-homologous recombination breakpoints at very similar positions in the SIR. We observed a very high density of breakpoints within an ∼25 bp region near the 5′ edge of the SIR (Fig. 6). This tight breakpoint cluster is immediately 5′ to the site at which a primer-like molecule binds to initiate complementary strand replication (Donson *et al.*, 1984).

Besides this obvious breakpoint cluster, the other detectable breakpoints also appear to be non-randomly distributed. The distribution has features in common with that of homologous recombination breakpoints seen in both the recombinants described here and viruses sampled from nature (Fig. 2).

## DISCUSSION

We used a simple experimental system to generate and select recombinant geminivirus genomes, and thereby demonstrated that recombination can be an extremely powerful mechanism enabling rapid adaptive evolution of MSV. The high resolution of this system, arising from the high density of informative sites that differentiate the known parental sequences, allowed us to accurately and precisely identify crossover events. Moreover, prior knowledge of at least one extremely fit ‘target recombinant solution’ (MSV-MatA), and the large fitness difference between this target solution and the parental sequences, allowed an assessment of the degree to which emergent recombinants approximated an ideal target solution. Our results show that even though recombination often resulted in surprisingly complex mosaic sequences, comprising up to nine alternating sections of parental DNA sequences in one case, the progeny genomes nevertheless contained an average of 91.7 % of the polymorphisms expected in the target solution.

### Complex recombinants might not be detectable in nature

Our discovery of complex recombinants emphasizes the importance of experimental analyses of recombination under controlled conditions. Considering the severe limitations of current computational tools for detecting recombination breakpoints, it is not surprising that complex recombinants like those described here have never been detected in nature. Even if exact parental DNA sequences were available in datasets sampled from nature, and if each recombinant were analysed separately (which strongly biases the analysis in favour of detecting recombination because no statistical corrections are needed for multiple testing), 22 of 32 recombination breakpoints in our complex recombinant sequences would remain undetected using the default settings of seven popular recombination detection methods implemented in the program RDP3 (Martin *et al.*, 2005b; Supplementary Fig. S1, available in JGV Online). Although these methods implied that recombination had occurred, only recombination breakpoints bounding large tracts of exchanged sequence were identifiable. While it is unknown whether complex MSV recombinants such as those we have described here occur and are successful in nature, such recombinants might critically affect sequence analysis techniques that are hindered by recombination. Such methods include, but are not limited to, phylogenetic tree construction (Posada & Crandall, 2002), molecular clock estimation (Schierup & Hein, 2000) and analysis of positive selection (Scheffler *et al.*, 2006).

### Selective processes strongly influenced the recombination patterns we observed

The strong selection pressures that were undoubtedly responsible for driving the rapid adaptation we observed in these recombination experiments, most likely also resulted in the loss of many recombinant genomes that were less fit than those that we ultimately recovered and characterized. The observed recombination breakpoint patterns were probably influenced by (i) variations in the underlying mechanistic rates at which recombination occurs across MSV genomes, (ii) the anatomy of the chimaeric input viruses and (iii) the sequence of the ‘target’ recombinant solution, MSV-MatA. The most simple and efficient process for obtaining MSV-MatA-like genomes from the input viruses would require the transfer of the entire *mp*–*cp* cassette from VWMPCPMat to MatMPCPVW via two crossover events. Indeed, the majority of the recovered recombinants appeared to have arisen through this type of exchange. Thus, when considering the frequency distribution of recombination breakpoints in our dataset, it is clear that selection has strongly favoured recombinants with breakpoints near the original junctions between wild-type sequences in the chimaeric parental viruses.

Nevertheless, the presence of significant proportions of MSV-VW DNA sequences in the *mp* of some recombinants indicates that the differential fitness of MSV-VW and MSV-MatA in maize is possibly only determined by a small number of sites within this protein (see the ‘conserved solution’ in Fig. 1). In support of this, Martin & Rybicki (2002) showed that substitution of the MSV-MatA *mp* with that of MSV-VW has only a slight impact on virulence. Conversely, this same study found that substitution of either *cp* or *rep* (*repA*+*repB*) of MSV-MatA with the corresponding MSV-VW genes severely crippled the resulting viruses. Accordingly, none of the recombinants characterized here contained any MSV-VW *rep* sequences (Figs 1 and 4), and all of them encoded CPs almost identical to that of MSV-MatA. This was at least partially due to MSV CP sequences being highly conserved, with the C-terminal third of the MSV-MatA and MSV-VW CPs sharing 100 % identity, despite differences in their coding sequences.

Our experimental system for generating recombinant viruses relied on selection for high fitness genomes, which imposes certain constraints on its use. As it is presented here, it can be used neither to quantify variations in the underlying mechanistic rates at which recombination occurs in different parts of virus genomes nor to accurately infer selective constraints on recombination breakpoints at particular sites. These limitations stem from the fact that natural selection is likely to favour recombinant genomes that resemble the ideal ‘target solution’ (in this case MSV-MatA). Quantification of relative mechanistic rates of recombination across the genome would require the complete removal of selection such that deleterious, advantageous and neutral recombinant events would be sampled with equal probability. Similarly, the characterization of fitness constraints on breakpoints at specific sites would require unbiased sampling of neutral and advantageous recombination events. Some of these problems could be addressed by progressively reducing the differences in fitness between chimaeras and their corresponding wild-type ‘target recombinant solutions’. Alternatively, less selective host genotypes could be used for co-infections. Nevertheless, we cannot suggest practical ways to entirely remove, or account for, the biasing effects of selection.

### Evidence for a mechanistic origin of recombination hotspots

Notwithstanding the unquestionable role that selection played in determining the configurations of the recovered recombinants, we found evidence of two mechanistically predisposed breakpoint hotspots in the MSV genome. Recombinant genomes isolated from multiple plants showed evidence of breakpoints at the v-*ori*. Similarly, many of the breakpoints that we observed in full-length genomes and in the rearranged subgenomic DNA fragments occurred within an ∼25 bp region in the SIR, between the *cp* stop codon and the probable binding site of the primer for complementary-sense replication. The v-*ori* hotspot has been previously detected in other experimental investigations of geminivirus recombination (García-Andrés *et al.*, 2007b; Schnippenkoetter *et al.*, 2001; Stenger *et al.*, 1991), and recombination hotspots at both the v-*ori* and at the 3′ end of *cp* have been detected in computational analyses of naturally sampled geminivirus genomes (Lefeuvre *et al.*, 2007a; Varsani *et al.*, 2008b).

Lefeuvre *et al.* (2007a) and Varsani *et al.* (2008b) attempted to explain these recombination hotspots by accounting for both selective and mechanistic factors. Specifically, it has been reasoned that the SIR/*cp* region is a recombination hotspot because (i) the SIR and the 3′ portion of *cp* may be relatively robust to the disruptive effects of recombination and (ii) the SIR may constitute a region in which frequent clashes occur between replication and transcription complexes, which are known to stimulate strand breakage and recombination (Huertas & Aguilera, 2003; Takeuchi *et al.*, 2003). However, the recombination breakpoint hotspot that we identified within a 25 nt region of the SIR, immediately 5′ of the site at which complementary strand MSV replication is primed, might imply that there is an additional (or alternative) mechanistic explanation for the hotspot observed in sequences sampled from nature. It is possible that either synthesis of, or DNA extension from, the RNA fragment that primes complementary strand synthesis may disrupt virion strand replication and contribute to the frequency of recombination in this region.

### The origin of complex recombinants

While the assembly of complex recombinants might occur via a series of independent, temporally separated recombination events spanning multiple replication cycles, it is also possible that they are produced by multiple crossovers occurring during single, repetitively interrupted, replication cycles. Amongst all the full-length and subgenomic genomes that we examined from individual plants, we found no clear evidence of intermediate forms that would have indicated that complex recombinants were assembled over multiple replication cycles. We detected only one instance where two recombinants isolated from the same plant differed with respect to a single pair of breakpoints (compare the LIRs of R4-01 and R4-46 in Fig. 1). However, the supposed recombination event differentiating these clones is suspicious, in that it was inferred from a single polymorphic site, suggesting that it might instead represent a single nucleotide substitution event.

One possibility is that simple and complex recombinants are alternative products of the breakage and repair of double-stranded, episomal, replicative form (RF) DNAs that occur during rolling circle replication (RCR). Following a double-strand break of a RF DNA, it would be expected that host-mediated recombinational repair would proceed via the recombination-dependent replication (RDR) pathway (Jeske *et al.*, 2001). However, depending on whether the complementary or the virion strand of the broken RF DNA is used to initiate the repair, two outcomes are possible. If it is the virion strand that primes RDR, then the repair would occur in the same direction as normal RCR. Under these conditions, simultaneous RCR and RDR could potentially proceed unhindered on the same molecule. Alternatively, if the complementary strand primes RDR, then the repair would proceed in the opposite direction to normal RCR and both processes might therefore be interrupted frequently. Repeated collisions between replication complexes moving in opposite directions might provide multiple opportunities for replication to be reinitiated using different template molecules each time. Recombinational repairs that are primed by the virion strand and proceed in the direction of replication could be expected to yield something resembling our simple recombinants (i.e. those with one breakpoint at the v-*ori* and another at the point where the repair was initiated), whereas repairs primed by complementary strands might be expected to yield something resembling complex recombinants such as R5, R6 and R11 (Fig. 1).

### The origin and significance of subgenomic DNAs

The frequency distribution of breakpoints in the subgenomic DNAs we have characterized is strikingly similar to that of subgenomics found in natural infections and of full-length recombinant genomes sampled from nature. Importantly, the increased prevalence of breakpoints in, or adjacent to, the SIR and near the v-*ori* have also been documented by Casado *et al.* (2004) using completely different MSV subgenomic isolation and cloning strategies to those used here. The study by Casado *et al.* (2004) also identified a large number of subgenomics with breakpoints within the same 25 nt SIR recombination hotspot we have identified. This clearly indicates that the non-random subgenomic breakpoint distributions we have observed are not simply an artefact of our experimental design.

The congruence between these distributions and those observed in natural recombinants implies that the same underlying processes are probably responsible for the generation of both full-length and subgenomic recombinants. High molecular mass linear DNA (hDNA) forms are apparently a ubiquitous feature of geminivirus replication (Saunders *et al.*, 1991; Preiss & Jeske, 2003) that are now believed to be the products of RDR pathways (Jeske *et al.*, 2001). The ends of these hDNA molecules in *Abutilon* mosaic virus (Jeske *et al.*, 2001) map most frequently to recombination hotspots detectable in other begomovirus genomes sampled from nature (Lefeuvre *et al.*, 2007a, b), indicating a clear link between RDR and recombination. Although the ends of MSV hDNA molecules have not been mapped, the similarity of breakpoint distributions in subgenomic and full-length DNAs possibly reflects the relative abundance of linear MSV hDNA molecules possessing ends within the SIR and at the v-*ori*. The difference between full-length recombinants and subgenomics may simply be that they are products of either homologous or non-homologous hDNA recombination, respectively.

### Conclusions

We have used an experimental, chimaera-based analysis system to demonstrate that recombination in geminiviruses can be both highly adaptive and more complex than has been previously appreciated. The occurrence of complex recombinants is interesting because the defective viruses we have used in these experiments could have potentially acquired very high fitness genotypes through only two crossover events. If the results of evolutionary protein engineering studies (Drummond *et al.*, 2005) can be extrapolated to virus genomes, it is probable that, in most instances at least, pairs of genetically distinct natural viruses might only be capable of producing higher fitness recombinants through more than two crossovers. In turn, this would suggest that there could be evolutionary pressures operating to optimize the numbers of crossovers that occur during recombination events. To test some of these possibilities, it would be of great interest to determine how effectively recombination can retrieve high fitness genomes from defective parental chimaeras with more than two breakpoints.

## Supplementary Material

[Supplementary Figure]
