# Supplementary material for: Rapid host adaptation by extensive recombination
Source: J Gen Virol. 2009 Mar;90(Pt 3):734–46. doi: 10.1099/vir.0.007724-0 (PMC2885065; doi:10.1099/vir.0.007724-0)
Supplement: [Supplementary Figure] [file supp_90_3_734__index.html]

 Rapid host adaptation by extensive recombination -- van der Walt et al. 90 (3): 734 Data Supplement - Supplementary Figure -- Journal of General Virology

### Rapid host adaptation by extensive recombination, by E. van der Walt, E. P. Rybicki, A. Varsani, J. E. Polston, R. Billharz, L. Donaldson, A. L. Monjane and D. P. Martin

*Journal of General Virology* vol. **90**, part 3, pp. 734 - 746

**Supplementary Fig. S1.** Recombination breakpoints detectable by one or more of seven commonly used recombination analysis methods implemented in the program RDP3. [PDF] (56 kb)

  
  
