# Supplementary material for: Rapid host adaptation by extensive recombination
Source: J Gen Virol. 2009 Mar;90(Pt 3):734–46. doi: 10.1099/vir.0.007724-0 (PMC2885065; doi:10.1099/vir.0.007724-0)
Supplement: [Supplementary Figure] [file supp_90_3_734__1.pdf]

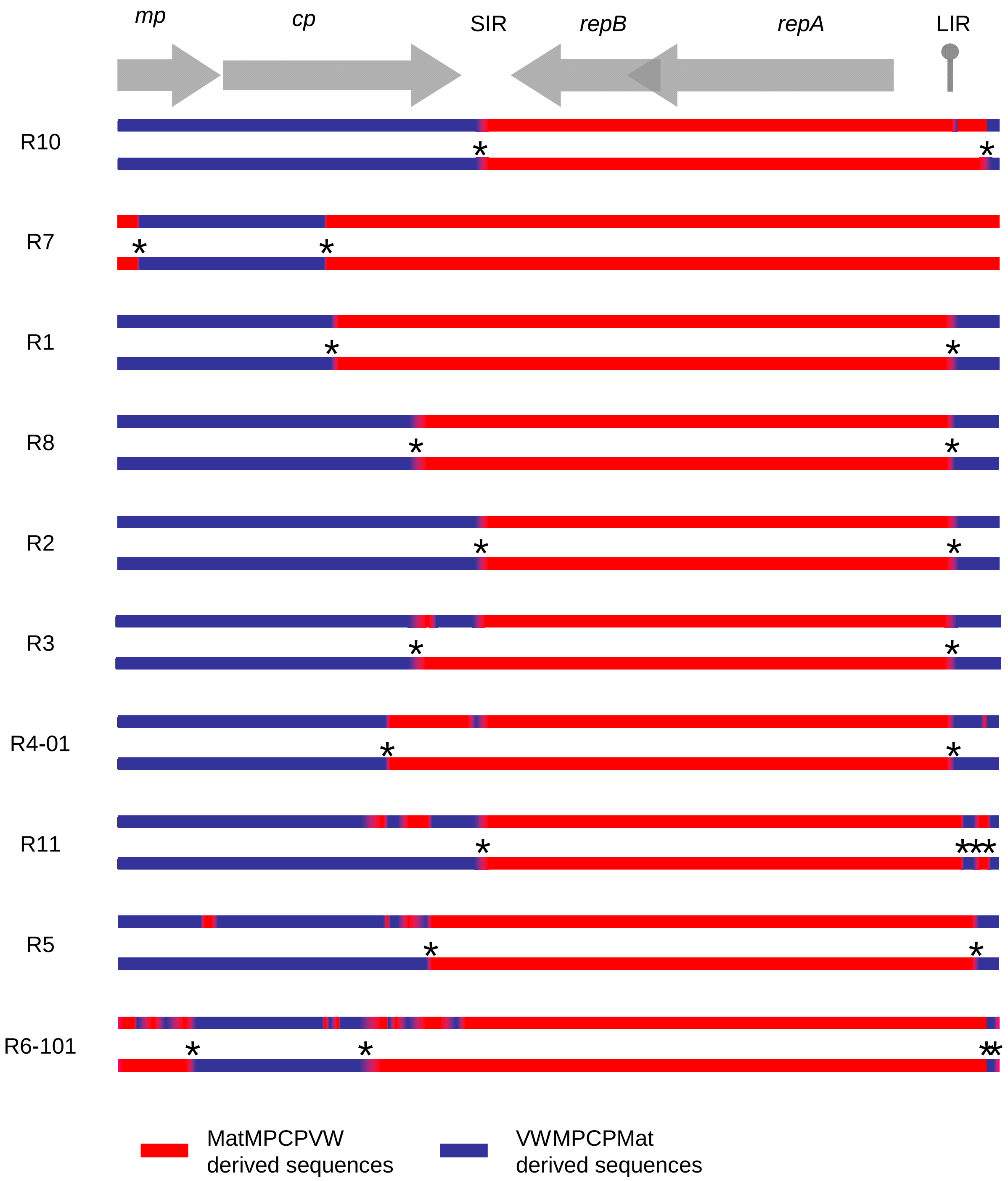


**Supplementary Fig. S1**. Recombination breakpoints detectable by one or more of seven commonly used recombination analysis methods implemented in the program RDP3 (Martin *et al.*, 2005). Genome regions (above) are labelled as described in Fig. 1 of the main text.

---

## Supplementary Reference

**Martin, D. P., Williamson, C. & Posada, D. (2005).** RDP2: recombination detection and analysis from sequence alignments. *Bioinformatics* **21**, 260–262.

---

**van der Walt, E., Rybicki, E. P., Varsani, A., Polston, J. E., Billharz, R., Donaldson, L., Monjane, A. L. and Martin, D. P. (2009).** Rapid host adaptation by extensive recombination. *J Gen Virol* **90**, 734–746.
